# Extra-Cranial Carotid Artery Stenosis: An Objective Analysis of the Available Evidence

**DOI:** 10.3389/fneur.2022.739999

**Published:** 2022-06-21

**Authors:** Anne L. Abbott

**Affiliations:** ^1^Department of Neuroscience, Central Clinical School, Monash University, Melbourne, VIC, Australia; ^2^Neurology Private Practice, Knox Private Hospital, Wantirna, VIC, Australia

**Keywords:** best practice, stroke prevention, carotid stenting, carotid stenosis, medical intervention, carotid endarterectomy, guideline standards, transcarotid arterial revascularisation

## Abstract

**Background and Purpose:**

Carotid stenosis is arterial disease narrowing of the origin of the internal carotid artery (main brain artery). Knowing how to best manage this is imperative because it is common in older people and an important cause of stroke. Inappropriately high expectations have grown regarding the value of carotid artery procedures, such as surgery (endarterectomy) and stenting, for lowering the stroke risk associated with carotid stenosis. Meanwhile, the improving and predominant value of medical intervention (lifestyle coaching and medication) continues to be underappreciated.

**Methods and Results:**

This article aims to be an objective presentation and discussion of the scientific literature critical for decision making when the primary goal is to optimize patient outcome. This compilation follows from many years of author scrutiny to separate fact from fiction. Common sense conclusions are drawn from factual statements backed by original citations. Detailed research methodology is given in cited papers. This article has been written in plain language given the importance of the general public understanding this topic. Issues covered include key terminology and the economic impact of carotid stenosis. There is a summary of the evidence-base regarding the efficacy and safety of procedural and medical (non-invasive) interventions for both asymptomatic and symptomatic patients. Conclusions are drawn with respect to current best management and research priorities. Several “furphies” (misconceptions) are exposed that are commonly used to make carotid stenting and endarterectomy outcomes appear similar. Ongoing randomized trials are mentioned and why they are unlikely to identify a routine practice indication for carotid artery procedures. There is a discussion of relevant worldwide guidelines regarding carotid artery procedures, including how they should be improved. There is an outline of systematic changes that are resulting in better application of the evidence-base.

**Conclusion:**

The cornerstone of stroke prevention is medical intervention given it is non-invasive and protects against all arterial disease complications in all at risk. The “big” question is, does a carotid artery procedure add patient benefit in the modern era and, if so, for whom?

## Introduction: Key Concepts

***Carotid stenosis*** refers to atherosclerotic narrowing of the origin of the internal carotid artery (the main brain artery). The scientific literature and guideline recommendations tend to focus on ***advanced (50–99% or 60–99%) carotid stenosis***. This follows from randomized trials that showed an overall stroke risk reduction benefit from surgery (carotid endarterectomy, CEA) compared to ***medical intervention*** alone (lifestyle coaching and medication). That surgical benefit was only seen in ***highly selected*** patients with at least 50 or 60% carotid stenosis (1). There has never been direct comparison of any other so-called **“*carotid revascularization” procedure*** with medical intervention alone. Stroke prevention benefit with respect to other procedures, such as carotid artery angioplasty with stenting (CAS), are based on the assumption that randomized trials of CEA vs. medical intervention alone (that were conducted 3–4 decades ago) are still applicable.

In this article the ***definition of stroke*** is generally one based on an appropriate neurological deficit lasting at least 24 h, and a ***transient ischaemic attack (TIA)*** lasting <24 h (2). The risk of ipsilateral (same-sided) stroke in individuals with 50–99% or 60–99% carotid stenosis is approximately double the risk for individuals with lesser stenosis (3, 4). The ***main method of measuring carotid stenosis*** in previous trials and guideline recommendations is derived from conventional intra-arterial angiography and the North American Symptomatic Carotid Endarterectomy Trial (NASCET): the ratio of residual lumen at the point of maximal stenosis to the distal lumen where arterial walls first become parallel) (4, 5). This method (or similar methods) will be referred to in this article unless otherwise stated. Most arterial imaging is now done non-invasively, and measuring carotid stenosis is not an exact science (6–10).

***Individuals with carotid stenosis are generally differentiated according to whether or*** ***not they have had previous stroke or TIA*** affecting the brain region or eye ipsilateral to (i.e., same-sided and, therefore, in the vascular territory of) the carotid stenosis. This distinction is made because carotid artery procedures are targeted interventions that focus on reducing the risk of stroke caused by the carotid stenosis. In addition, symptomatic patients have a much higher short-term stroke rate than asymptomatic patients and are more likely to benefit from CEA (4, 11–13).

The term **“*symptomatic carotid stenosis”*** has been widely used. However, it is a misnomer. It is inherently procedurally biased because it implies that the carotid stenosis was responsible for the stroke or TIA in any given patient. This implication of causality is inappropriate given that ~50% of symptomatic individuals with ipsilateral carotid stenosis have another readily identifiable possible cause of their stroke or TIA and that cannot be treated by a carotid artery procedure (3). Thus, the presence of a condition, such as carotid stenosis, does not mean causation (14). The correct terminology, which encourages us to think holistically, is a ***symptomatic individual with ipsilateral carotid stenosis*** (ipsilateral to the affected brain region or eye).

Further, ***any stroke risk reduction benefit seen in past randomized CEA trials was an*** ***overall benefit***. Some patients were harmed, most did not benefit and some with asymptomatic carotid stenosis would have been included in analyses of symptomatic patients. In any given patient, it is usually impossible to be certain of the cause of stroke or TIA. Risk factors and probabilities are more applicable in diagnosis and management (2). By contrast, a person with ***asymptomatic carotid stenosis*** has never before had a clinically-defined ipsilateral stroke or TIA (2). However, such an individual may not be asymptomatic with respect to the rest of their brain or arterial system. Asymptomatic carotid stenosis may exist in a completely asymptomatic person or a symptomatic person with respect to past clinically manifest arterial disease complications.

***Advanced asymptomatic carotid stenosis is common in older people***, affecting about 10% of individuals by their eighth decade (13). Advanced carotid stenosis is easily detected using non-invasive imaging and causes about 10% of all strokes (13). Carotid stenosis also identifies individuals at higher risk of other preventable arterial disease complications, such as myocardial infarction (15–17). Therefore, knowing how to manage this lesion is very important.

***Medical intervention is indicated for all individuals with carotid stenosis*** whether or not they have a carotid procedure. In contrast to carotid procedures, medical intervention reduces the risk of all arterial disease complications, including all stroke (ischaemic and haemorrhagic) and TIA affecting all parts of the brain, by addressing risk factors such as hypertension, hypercholesterolaemia, tobacco smoking, atrial fibrillation, diabetes, excessive weight and alcohol consumption and physical inactivity.

Medical intervention is fundamentally the same in individuals whether or not they have carotid stenosis. Therefore, determining current best medical intervention for carotid stenosis patients has implications for best preventing all arterial disease complications and best protecting all individuals with arterial disease risk. This is very important given that arterial disease is the lead cause of death worldwide and a leading cause of premature death and disability with huge social and economic consequences (18–20).

The stroke risk reduction benefit from medical intervention alone in carotid stenosis patients has improved significantly over the last 3–4 decades since past randomized comparisons with CEA were performed and is very effective (13, 21–28). ***The “big”*** ***question is***, **“*Can a carotid artery procedure provide an additional stroke risk reduction*** ***benefit compared to current best practice medical intervention alone?”*** The evidence-base regarding each interventional approach for carotid stenosis will now be presented and discussed.

## Interventions For Carotid Stenosis And Reducing Stroke Risk

Currently there are four types of intervention done in the name of reducing stroke risk associated with carotid stenosis:

*i*. ***Carotid surgery or endarterectomy*** (CEA, surgical removal of the atherosclerotic plaque causing stenosis).
*ii*. ***Carotid angioplasty with stenting*** (CAS, balloon dilation of the stenosis, and stent placement *via* an intra-arterial catheter).
*iii*. A new CEA-CAS hybrid-type procedure, known as ***trans-carotid arterial*** ***revascularisation** (TCAR)*.
*iv*. ***Medical intervention*** (risk factor identification and lifestyle coaching/healthy lifestyle habits and appropriate medication).

Worldwide, carotid artery procedures make up a multi-billion dollar per year international industry. Table 1 shows published data from just a few countries with respect to use of CEA and CAS. The estimated procedural costs are based on 2007 estimates from the United States of America. There is notable heterogeneity in target populations for CEA and CAS between countries, as well as between hospitals within countries with respect to patient symptomatic status, age and sex (29). It is perhaps surprising to see such heterogeneity, given that all procedural centers should have access to the same evidence-base. Procedural intervention is more common in countries with “fee for service” reimbursement' where physician payment is proportional to the number of procedures performed (29).

**Table 1:** The multi-billion dollar per year global carotid procedural industry (29–34).

**Country**	**Procedures done/Year**	**Asymptomatic stenosis patients (%)**	**Procedural cost**	**Procedural complication cost^**∧**^**
United States of America	135,000*	92	2.7 billion	+ More
Germany	33,000*	57	0.7 billion	+ More
United Kingdom	5,700**	15	0.1 billion	+ More
Australia	3,200*	0–79	64 million	+ More
Total			3.6 billion	+ More

^*^CEA & CAS; ^**^CEA only; 2005–2007 US estimates for procedural cost: $20,000/CEA, $35,000/stent. ^∧^Complications such as death (~1%), stroke (~1–10%) and myocardial infarction (~1%).

### Carotid Endarterectomy

#### Patients With Advanced Asymptomatic Carotid Stenosis

We have known for a long time from randomized trial evidence that CEA is inefficient for reducing stroke risk associated with advanced asymptomatic carotid stenosis (12). The Asymptomatic Carotid Atherosclerosis Study (“ACAS”) remains the largest randomized trial of medical intervention plus CEA compared to medical intervention alone in patients with asymptomatic carotid stenosis (35). The Asymptomatic Carotid Surgery Trial (“ACST-1”) is sometimes mentioned in this context (36, 37). However, ACST-1 was a randomized trial of early vs. deferred CEA with no medical-intervention-only-arm. In ACST-1, 24% of patients allocated deferred CEA had CEA by study end. In 2004 it was reported that the peri-operative rate of stroke or death in patients who had “deferred” CEA was 4.5% (36). Further, ACST-1 included patients who had been symptomatic more than 6 months before recruitment (making up 12% of participants). Ipsilateral stroke (the most relevant outcome with respect to carotid artery procedures) was not an outcome measure in ACST-1. The “SPACE-2” Trial was initiated to compare outcomes with medical intervention with or without additional CEA or CAS in asymptomatic carotid stenosis patients. However, it was stopped early due to slow recruitment (38). Meanwhile, the Veteran's Affairs Cooperative Study was a randomized trial only involving men and it was underpowered with respect to stroke as an outcome measure (39).

**Figure 1:**
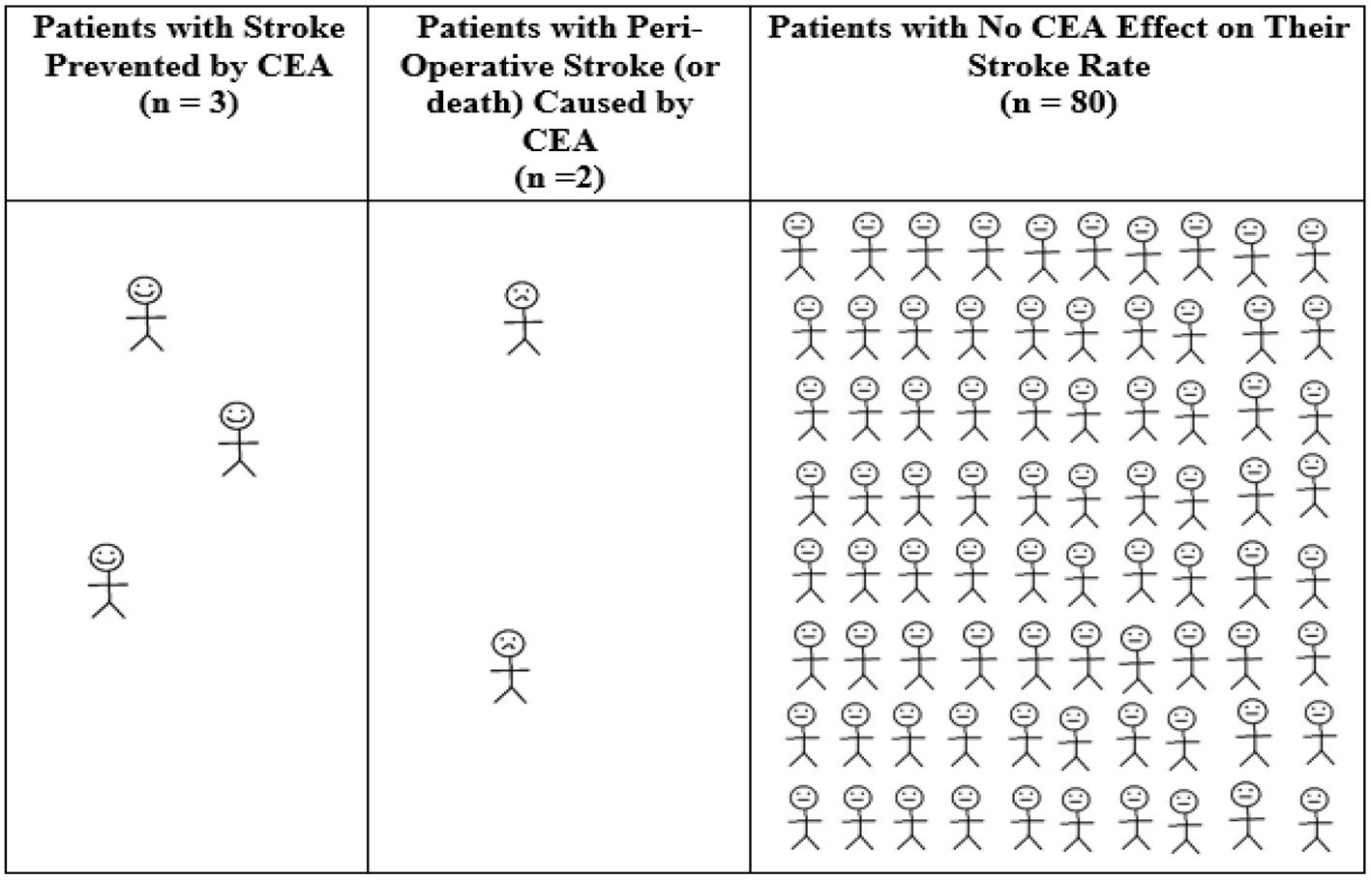
Average 12-month outcomes for every 85 patients with asymptomatic carotid stenosis randomized to CEA in ACAS (35). Calculated from ACAS data regarding patients with 60–99% asymptomatic carotid stenosis (using “NASCET” criteria), the overall absolute annual ACAS stroke risk reduction from CEA and the 2.3% 30-day CEA rate of stroke or death in ACAS (35). There was a 5.9% reduction in any peri-operative stroke/death or later ipsilateral stroke in ACAS with CEA over 5 years using Kaplan Meier analysis (11.0% with medical intervention alone vs. 5.1% with CEA, or 1.18%/year risk reduction with CEA). Therefore, the number needed to treat by CEA to be ahead by 1 ipsilateral stroke over 12 months of study follow-up was 100/1.18 = 85. Derived from a figure originally published in Fast Facts, 2012 (©S. Karger AG, Basel) (40).

Therefore, ACAS remains the main trial for purported justification for CEA in patients with asymptomatic carotid stenosis. Patients were randomized into ACAS between 1987 and 1993, ~3 decades ago. For every 85 patients with 60–99% asymptomatic carotid stenosis randomized to endarterectomy in ACAS, on average 3 patients had an ipsilateral stroke prevented over the next 12 months (see Figure 1) (35). However, that was at the expense of 2 patients who had a peri-operative stroke (or less commonly, peri-operative death). For the remaining 80 patients, CEA had no effect on their stroke risk over the next 12 months (35). The mean baseline age of ACAS participants was 67 years. They were all reasonably medically fit, as they were considered to be at low or average risk of major CEA complications and at low risk of death within the next 5 years.

Most patients did not have a stroke during follow-up in ACAS. For example, 89% of patients with 60–99% asymptomatic carotid stenosis in ACAS who were given medical intervention alone had not had an ipsilateral stroke by 5 years of projected follow-up (35). Only one subgroup of asymptomatic (or recently asymptomatic) carotid stenosis patients have ever been shown to have an overall statistically significant stroke reduction benefit from CEA. Using data from ACAS (with respect to CEA vs. medical intervention alone) and ACST-1 (with respect to early vs. deferred endarterectomy), it was only ***men aged*** **<*75–80*** ***years with 60–99% stenosis*** (using conventional intra-arterial angiography or ultrasound and NASCET criteria) who benefited. In addition, such men had to be free of any major life-threatening condition, have a life expectancy of at least 5 years and satisfy all trial selection criteria. The overall stroke prevention benefit for these men was small, ~1%/year (35–37). Women did not benefit from CEA in ACAS. Women coming closest to an overall stroke risk reduction benefit from early CEA compared to deferred CEA in the ACST-1 were aged <75 years. However, the result just failed to reach statistical significance (37).

The overall 30-day peri-operative rate of stroke or death was 1.7% in ACAS when the angiographic stroke risk was excluded and 2.3% when the angiographic risk was included (35). The overall 30-day peri-operative rate of stroke or death in ACST-1 was 3.1% (36, 37). Arguably, because ACAS was the most relevant trial, it should have been used for procedural hazard standards in routine practice. In addition, since the publication of ACAS in 1995, conventional intra-arterial angiography has not been accepted as best practice for identifying or assessing patients with asymptomatic carotid stenosis. It has generally been replaced by safer non-invasive methods (41). Hence, it could be argued that a 30-day peri-operative rate of stroke or death of 1.7% (rather than the generally accepted 3% rate) should have been used in routine practice as the standard for inferring an overall procedural benefit for asymptomatic carotid stenosis patients (1, 12).

Adding to evidence of net patient harm in routine practice, CEA outcomes in routine practice (when measured) are often, if not usually, worse compared to those seen in ACAS or ACST-1 (13, 42, 43). For example, in a meta-analysis of 30-day stroke or death rates associated with CEA in 47 high volume registries of asymptomatic carotid stenosis patients, it was only by about 2003 (8 years after ACAS was published) that the average 30-day CEA stroke or death rate in those registries averaged 2.3%, and only by about 2010 (15 years after ACAS was published) that this rate averaged 1.7% (42). Moreover, and as explained below, 30-day stroke or death rate standards derived from ACAS and ACST-1 have been increasingly outdated and excessive since they were published due to ongoing improvements in the stroke prevention effectiveness of medical intervention alone (12).

#### Symptomatic Patients With Advanced Carotid Stenosis

It has been known for a long time from randomized trial evidence that CEA is inefficient for stroke risk reduction in symptomatic patients with advanced ipsilateral carotid stenosis. There were 3 sufficiently large randomized trials of medical intervention with or without additional CEA to be impactful on routine practice: Veterans Affairs 309 Trial (VA309), North American Symptomatic Carotid Endarterectomy Trial (NASCET) and European Carotid Surgery Trial (ECST) (44–46). Patients were randomized into these trials between 1981 and 1994, ~3–4 decades ago. Symptomatic patients with advanced carotid stenosis were more likely to benefit from CEA in randomized trials than patients with advanced asymptomatic carotid stenosis. However, the overall ipsilateral stroke risk reduction benefit for symptomatic patients with 70–99% carotid stenosis (using NASCET criteria and in the absence of near occlusion) was modest, 3.2%/year (4). Most patients did not have a stroke during follow-up in these trials. For example, 74% of symptomatic patients with 70–99% stenosis in NASCET who were given medical intervention alone had not had an ipsilateral stroke by 2 years of follow-up (47).

**Figure 2:**
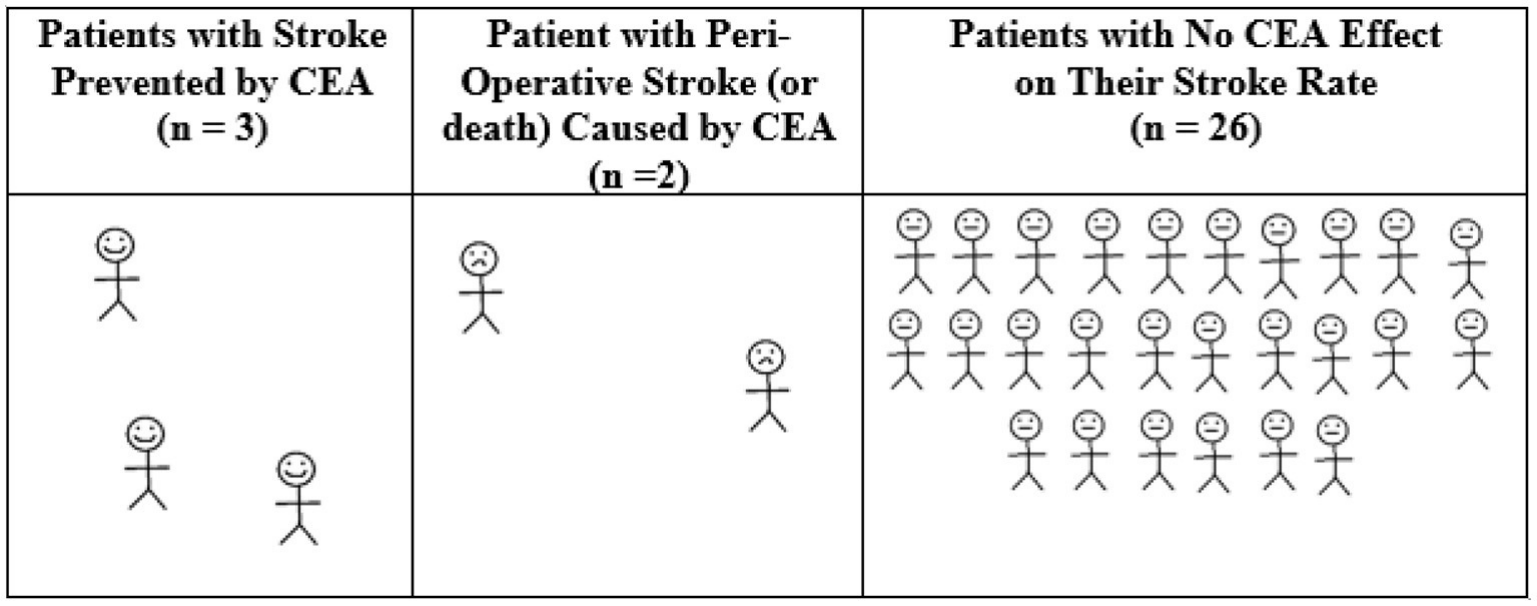
Average 12-month outcomes for every 31 symptomatic patients randomized to CEA in NASCET, ECST, and VACS. Calculated from pooled randomized trial data regarding symptomatic patients with 70–99% stenosis (using “NASCET criteria” and excluding near occlusion), the overall absolute stroke risk reduction with CEA and the overall 30-day CEA rate of stroke or death of 6.2% (4). There was a 16% reduction in any peri-operative stroke/death or later ipsilateral stroke with CEA over 5 years using Kaplan Meier analysis (~27.0% with medical intervention alone vs. 11.0% with CEA, or 3.2%/year risk reduction with CEA) (4). Therefore, the number needed to treat by CEA to be ahead by 1 ipsilateral stroke over 12 months of study follow-up was 100/3.2 = 31.25 (40). Derived from a figure originally published in Fast Facts, 2012, and now with a correction (©S. Karger AG, Basel) (40).

Using pooled data (4), for every 31 patients randomized to CEA across NASCET, ECST, and VA309, on average 3 patients had a stroke prevented over the next 12 months of follow-up (see Figure 2). However, that was at the expense of 2 patients who had a peri-operative stroke (or less commonly, peri-operative death). For the remaining 26 patients, CEA had no effect on their stroke rate over the next 12 months (40). The average baseline age of participants in these trials was 60–66 years. All were reasonably medically fit, as they were considered to be at low or average risk of major CEA complications and at low risk of death within the next 3–5 years.

Very few subgroups of symptomatic patients were shown to have a statistically significant (overall) benefit with respect to reduced stroke rate from CEA in these randomized trials. They satisfied all the trial selection criteria, had a life expectancy of at least 3–5 years, did not have near carotid occlusion [angiographic evidence of severe stenosis and reduced distal flow (4)] and fit into one of these three groups:

*i*. ***Women with 70–99% stenosis*** *(by way of conventional intra-arterial angiography and NASCET criteria)* having CEA ***within 2–3 weeks*** of their last same-sided stroke or TIA.
*ii*. ***Men with 50–69% stenosis*** *(by way of conventional intra-arterial angiography and NASCET criteria)* having CEA within ***2–3 weeks*** of their last same-sided stroke or TIA.
*iii*. ***Men with 70–99% stenosis*** *(by way of conventional intra-arterial angiography and NASCET criteria)* having CEA within ***3 months*** of their last same-sided stroke or TIA. However, the benefit fell rapidly over this time and was highest ***within 2–3 weeks*** of their last same-sided stroke or TIA (1, 48).

Overall, in these randomized trials, the 30-day peri-operative rate of stroke or death associated with CEA was ~6%. This is the standard that has been used in routine practice to infer an overall CEA benefit for symptomatic patients with advanced carotid stenosis compared to using medical intervention alone. However, because of advances in medical intervention since these randomized trials were conducted, 6% is now excessive as a procedural standard, fewer symptomatic patients are now likely to benefit from CEA and the window of opportunity for procedural benefit might now be shorter (49). New randomized trials of CEA vs. medical intervention alone in suitable symptomatic patients are a priority (see below).

#### Patient Subgroups and CEA Harm

Different sample sizes, patient selection criteria, patient risk factor profiles, procedural risks, standards of medical intervention, follow-up duration, definitions, and reporting methods cause heterogeneity between studies in detecting significant subgroup treatment differences.

##### Information From Individual Randomized Trials

Past randomized trials have shown that ***symptomatic patients*** are more likely to suffer peri-operative stroke or death than patients with ***asymptomatic*** carotid stenosis (see Figures 1, 2) (50). Further, ***women*** were more likely to experience stroke or death after CEA than men in ACAS and ACST-1. However, these trials were not sufficiently powered to test the influence of patient sex on procedural complication rates (they had sample sizes of 1,662 and 1,320, respectively) (35–37). These trials were similarly underpowered to test the effect of ***age*** on procedural risk. The mean baseline age in ACAS and ACST-1 was 67 and 68 years, respectively. Patients over 80 years were excluded from ACAS and patients over 75 years at baseline comprised only 650 of all patients randomized in ACST-1 (36).

***Symptomatic women*** had a significantly higher peri-operative rate of stroke or death in the pooled analysis of NASCET and ECST (OR 1.50, 95% CI 1.14–1.97, *P* = 0.04, 5,893 total patients) (51). Using the same pooled data, a higher peri-operative rate of stroke or death was not found according to ***age*** (<65, 65–74, and >75 years) (51). However, the average baseline age of patients in NASCET and ECST was 66 and 63 years, respectively (45, 46). There were only 409 (14.2%) NASCET and 176 (5.9%) ECST patients aged ≥75 years at baseline, indicating under powering for the analysis of age as a risk factor for CEA complications (51).

##### Information From Meta-Analyses of Randomized and Non-randomized Studies

In a meta-analysis of 25 studies of mixed symptomatic and asymptomatic patients, Bond et al. documented ***women*** had a higher rate of peri-operative stroke and death with CEA than men (OR 1.31, 95% CI = 1.17–1.47, *P* < 0.0001) (52). Bond et al. also reported a higher operative mortality in combined male and female patients of mixed symptomatic status ***aged*** **≥** ***80 years*** compared to younger patients in a meta-analysis of 15 studies (OR = 1.80, 95% CI = 1.26–2.45, *P* < 0.001) (52). The authors noted that, unfortunately, there were too few reports in the literature that stratified outcomes by both sex and symptomatic status for detailed patient subgroup analyses (52).

Since then, registries have provided outcome data for larger samples. For example, multivariable regression from the Nationwide German Statutory Quality Assurance database of 142,074 CEAs done for asymptomatic and symptomatic patients from 2009 to 2014 showed that more ***advanced age*** was associated with a higher procedural rate of any stroke or death until discharge (relative rate per 10 year increase 1.19 between ages <65 and ≥80 years; 95% CI 1.14–1.24) (50). Meanwhile, Khatri et al. found that ***age*** **≥*70*** ***years*** was a predictor of stroke, mortality and cardiac complications after both CEA and CAS in a multivariate analysis of 495,331 patients of mixed symptomatic status included in the Nationwide Inpatient Sample database (NIS) between 2005 and 2008 (OR 1.3 for both procedures, 95% CI 1.1–1.7 for CAS and 1.2–1.4 for CEA) (53). ***Female sex*** was not associated with a significantly increased CEA peri-procedural risk in the German database analysis or a 2000–2009 analysis from the NIS involving 221,253 CEA patients (50, 54). However, female sex was associated with a higher rate of peri-operative mortality in analysis of 21,597 symptomatic patients in the Vascular Quality Initiative (55).

### Carotid Angioplasty and Stenting

CAS was introduced as a less invasive alternative to CEA. However, it is clear that CAS (by the transfemoral/transaortic approach) is more dangerous for patients than CEA. CAS has not yet been compared to any standard of medical intervention in a trial. However, the SPACE-2 Trial was a notable missed opportunity and new randomized trials are underway with this objective (see below) (38). Past randomized trials of CEA vs. CAS (none of which included a medical-intervention-only treatment arm) and recommendations for CAS have evidently been based on the misconception that medical intervention has not changed since the earlier randomized trials of medical intervention with or without additional CEA. In every adequately powered randomized trial comparison, CAS was associated with about 1.5–2.0 times as many peri-procedural strokes or deaths as CEA (see below) (12, 49, 56, 57). This excess CAS peri-procedural rate of stroke or death is also seen in meta-analyses of randomized trials, registries and administrative databases, and it is not compensated by the peri-procedural rate of clinically-defined myocardial infarction (see below) (12, 31, 43, 49, 56–63).

Severe carotid re-stenosis is also more common after CAS than CEA and CAS tends to cost more (31, 56, 62). Complications (apart from stroke and death) that are more likely with, or particular to, CAS compared to CEA include hemodynamic instability (severe hypotension or bradycardia, including the need for a permanent pacemaker) and retroperitoneal hemorrhage (64–66). Cranial nerve injury and myocardial infarction are less common with CAS than CEA. However, overall in past randomized trials, peri-procedural stroke, death, and clinically defined myocardial infarction were more common after CAS than CEA (see below) (12, 56, 60, 65). Differing results have been reported with respect to “protection devices” for lowering the CAS associated peri-procedural rate of stroke or death (56, 67).

#### CAS and Patients With Asymptomatic Carotid Stenosis

Table 2 summarizes the results of randomized trials of CAS compared to CEA in patients with advanced asymptomatic carotid stenosis (or at least asymptomatic in the 6 months prior to randomization) with a sample size of >200 (range: 237–3,625 total subjects per trial). Each of these trials was underpowered to exclude a clinically significant difference in the peri-procedural rate of stroke or death between CAS and CEA, as indicated by 95% confidence intervals (CIs) which overlap 1. These trials include the second Asymptomatic Carotid Surgery Trial (ACST-2) (68). The 95% CIs in these trials extend to 4.9. This means that with a larger sample size, as would occur if the randomized trial methods were rolled out into routine practice, it is within the realms of probability that CAS could cause up to 4.9 times as many peri-procedural strokes or deaths as CEA in patients with asymptomatic carotid stenosis. Such adverse outcomes with CAS would be clinically significant.

**Table 2:** Peri-procedural rate of stroke or death with CAS compared to CEA in the largest randomized trials of patients with asymptomatic carotid stenosis.

**Randomized** **trial**	***n***	**Follow-Up (years)**	**30-Day** **peri-** **procedural stroke/death** **rate (%)**		**CAS** **excess**	***P***
			**CAS**	**CEA**	**OR/HR,** **95% CI**	
ACST-2 (68)^∧^	3,625	5 mean	3.5	2.6	OR 1.35 (0.91–2.03)*	0.12
ACT-1, 2016 (69)	1,453	0–5	2.9	1.7	OR 1.69 (0.70–4.10)*	0.24
CREST-1, 2010 (65)	1,181	2.5 median	2.5	1.4	HR 1.88 (0.79–4.42)	0.15
SPACE-2, 2016 (38)	400	1–5	2.5	2.0	OR 1.30 (0.34–4.90)*	0.70
SAPPHIRE, 2004 (70)	237	1	5.4	4.6	1.2 times higher (no raw data published to allow statistical calculations)	?

*Individually each randomized trial was statistically **underpowered** to exclude a clinically significant difference in the rate of peri-procedural stroke or death with CEA and CAS*.

^*^OR calculated from published raw data.

*^∧^Analysis using intention to treat figures*.

The direction of effect in each of these randomized trials was for 1.3–1.9 times as many 30-day peri-procedural strokes or deaths with CAS compared to CEA. This excess CAS harm in asymptomatic carotid stenosis patients reached statistical significance in a 2019 meta-analysis of randomized trials by Batchelder et al. (57) (7 randomized trials and 3,467 asymptomatic carotid stenosis patients). Registries and administrative databases of procedures performed on asymptomatic carotid stenosis patients also show excess peri-procedural rates of stroke and/or death with CAS compared to CEA (31, 43, 59). This excess procedural rate with CAS compared to CEA in asymptomatic carotid stenosis patients is consistent with the more clearly demonstrated statistically significant excess CAS stroke rate in randomized symptomatic patients (see below). For a given sample size, it is easier to show statistically significant differences in symptomatic compared to asymptomatic patients because of higher event rates in the symptomatic patients (71).

#### CAS and Symptomatic Patients With Carotid Stenosis

Table 3 summarizes the results of randomized trials of CAS compared to CEA in symptomatic patients with advanced carotid stenosis with a sample size of >200 (range: 219–1,713 total subjects per trial). Four of these trials were adequately powered to compare the peri-procedural rate of stroke or death between the procedures (as indicated by bold font in Table 3) (65, 72, 74, 76). These 4 trials showed that CAS was associated with approximately twice as many peri-procedural strokes or deaths as CEA. The direction of effect was similar in the underpowered trials. The comparison reached statistical significance in meta-analyses of randomized trials (56, 57). Registries and administrative databases of procedures performed on symptomatic patients with carotid stenosis likewise show higher peri-procedural rates of stroke and/or death with CAS compared to CEA (31, 43, 59).

**Table 3:** Peri-procedural rate of stroke or death with CAS compared to CEA in the largest randomized trials of symptomatic patients.

**Randomized trial**	***n***	**30/120 day** **stroke or** **death rate (%)**		**CAS excess OR/HR/RR, 95% CI**	***P***
		**CAS**	**CEA**		
**ICSS, 2010**^**∧**^ **(****72****)**	**1,713**	**8.5**	**4.7**	**HR 1.86 (1.26–2.74)**	**0.001**
**CREST-1, 2010** **(****65****)**	**1,321**	**6.0**	**3.2**	**HR 1.89 (1.11–3.21)**	**0.02**
SPACE-1, 2006 (73)	1,183	7.7	6.5	OR 1.19 (0.75–1.92)*	0.4
**EVA-3S, 2006** **(****74****)**	**527**	**9.6**	**3.9**	**RR 2.5 (1.2–5.1)**	**0.01**
CAVATAS, 2010# (75)	504	10.0	10.0	OR 1.00 (0.56–1.81)*	0.98
**Wallstent, 2001** **(****76****)**	**219**	**12.1**	**4.5**	OR 3.00 (1.01–8.61)*	**0.046**

*Bolded font indicates trials with sufficient statistical power to compare the peri-procedural rate of stroke and death with CEA and CAS*.

*^∧^120 day event rates*.

*#90% were symptomatic*.

^*^OR calculated from published raw data.

#### The Harm (Not Benefit) From CAS Is Durable

Table 4 summarizes the results of randomized trials of CAS vs. CEA in ***asymptomatic and/or symptomatic patients*** with a sample size of >400 and participant follow-up of ≥12 months. In every adequately powered comparison (as indicated in bold font) stroke in the longer term was significantly more prevalent in patients who had undergone CAS compared to CEA. As seen above, the results of these randomized trials show that patients experience more strokes and deaths in the ***peri-procedural period*** with CAS compared to CEA. However, rates of stroke ***beyond the peri-procedural period*** were similar with CAS and CEA. This observation, regarding the post-procedural period, is important because it means that most patients experiencing peri-procedural stroke live long-term with their strokes. It is inappropriate to exclude peri-procedural complications when making treatment choices (see below).

**Table 4:** Longer-term outcomes with CAS compared to CEA in the largest randomized trials of symptomatic and/or asymptomatic patients.

**Randomized trial**	***n***, **symptomatic status**	**Follow-up (years)**	**Outcome** **measure (%)** **CAS vs. CEA**		**CAS excess: HR/OR & 95% CI**	***P***
			**30-Day peri-procedural stroke/death or later ipsilateral stroke**			
**CREST-1, 2010** **(****65****)**	**2,502 SPts** **+** **APts**	**4 by KMA (median 2.5)**	**6.2**	**4.7**	**HR 1.44 (1.00–2.06)**	**0.049**
CREST-1, 2010 (65)	1,181 APts	4 by KMA (median 2.5)	4.5	2.7	HR 1.86 (0.95–3.66)	0.07
CREST-1, 2010 (65)	1,321 SPts	4 by KMA (median 2.5)	8.0	6.4	HR 1.37 (0.90–2.09)	0.14
**ICSS, 2015** **(****77****)**	**1,710 SPts**	**5 by KMA (median 4.2)**	**11.8**	**7.2**	**HR 1.72 (1.24–2.39)**	**<0.01**
SPACE-1, 2008 (78)	1,214 SPts	2 by KMA	9.5	8.8	HR 1.10 (0.75–1.61)	0.62
**EVA-3S, 2008** **(****79****)**	**527 SPts**	**4 by KMA (median 3.5)**	**11.1**	**6.2**	**HR 1.97 (1.06–3.67)**	**0.03**
			**30-Day peri-procedural death or any stroke**			
ACST-2 (68)^∧^	3,625 APts	5 mean	8.6	7.1	OR 1.23 (0.96–1.59)*	0.09
**CREST-1, 2016** **(****80****)**	**1,607 SPts** **+** **APts**	**10 by KMA (7.4 median)**	**11.0**	**7.9**	**HR 1.37 (1.01–1.86)**	**0.04**
CAVATAS, 2009# (81)	504 SPts + APts	8 KMA (median 5)	29.7	23.5	HR 1.35 (0.94–1.93)*	0.10
			**Any stroke free survival** ^**#**^			
ACT-1, 2016 (69)	1,453 APts	5 by KMA (median/mean not published)	93.1	94.7	Insufficient raw data published to calculate HR/OR

*KMA, Kaplan Meier analysis; SPts, symptomatic patients with advanced ipsilateral carotid stenosis; APts, patients with advanced asymptomatic or recently (for at least 6 months) asymptomatic carotid stenosis*.

*Bolded font indicates trials with sufficient statistical power to compare longer term stroke and death rate with CEA and CAS (including and beyond the peri-procedural period)*.

*#90% were symptomatic*.

^*^OR calculated from published raw data.

*^∧^Analysis using intention to treat figures*.

*#These figures on any stroke free survival in ACT-1 appear to exclude 30-day peri-procedural strokes (69)*.

There has been no adequately powered randomized trial of ***long-term outcomes of*** ***CAS compared to CEA in patients with asymptomatic carotid stenosis alone***. Underpowered trials include ACST-2 (68). However, as seen in Table 4, in an analysis from CREST-1 (comprising the 1607 asymptomatic and symptomatic patients remaining in follow-up), the 10 year rate of peri-procedural death or any stroke was significantly higher with CAS (11.0%) compared to CEA (7.9%): HR 1.37, 95% CI 1.01–1.86, *P* = 0.04) (80). This finding was consistent with the excess peri-procedural rate of stroke or death with CAS in CREST-1. Considering all CREST-1 2502 asymptomatic and symptomatic patients, the peri-procedural rate of stroke or death was significantly higher with CAS (4.4%) compared to CEA (2.3%): hazards ratio [HR] 1.90, 95% CI 1.21–2.98, *P* = 0.005 (65).

As also seen in Table 4, the 2006 EVA-3S randomized trial (79) and 2014 ICSS (International Carotid Stenting Study) (77) of ***symptomatic patients with carotid stenosis***, each found a statistically significant higher rate of ***peri-procedural stroke or death or later*** ***ipsilateral stroke*** at median 4.2 and 3.5 years of follow-up, respectively, with CAS compared to CEA. A higher rate of peri-procedural stroke or death or later ipsilateral stroke during study follow-up with CAS compared to CEA in symptomatic patients was also seen in a meta-analysis of randomized trials (OR: 1.59, 95%CI 1.16–2.16 in trials with the longest follow-up) (56).

#### Patient Subgroups and CAS Harm

CAS has *not* been shown to be more beneficial than CEA or medical intervention alone in any subgroup of carotid stenosis patients. In fact, as mentioned above, in every adequately powered randomized trial comparison to CEA, CAS caused significantly more peri-procedural strokes (with or without peri-procedural deaths or myocardial infarctions) and was associated with more strokes in the long-term (12, 49, 56, 57).

Particularly vulnerable to the stroke risk of CAS compared to CEA are:

- ***The most senior patients (aged*** **≥** ***70 years)***. Randomized trial comparisons in younger patients have been underpowered (53, 56, 60, 82–85).
- ***Those who are most recently symptomatic (especially within the previous 7–14*** ***days***, which is when best practice CEA is most likely to be beneficial) (63).
- ***Women*** (55, 56, 86, 87). However, men are also at higher risk of stroke or death from CAS compared to CEA (56).
- ***Those with certain carotid anatomical features***, such as longer, angulated, or tandem lesions (67, 88, 89).
- ***Those who have CAS in low volume centers or outside trials*** (56, 90, 91).

### Trans-carotid Arterial Revascularisation

There is a push to roll out a new hybrid procedure called **“*trans-carotid artery*** ***revascularization”*** (TCAR) into routine practice, despite no comparisons with current medical intervention alone and an unlikely benefit for at least the vast majority of patients (12, 92, 93). Currently, TCAR is only being assessed in registries and compared against CEA and transfemoral/transaortic stenting. Presently, there are no randomized trials comparing TCAR with current best medical intervention alone. However, ***a routine practice*** ***indication for TCAR (or any other arterial disease procedure) cannot be established*** ***without first showing that the procedure provides additional patient benefit compared*** ***to current best medical intervention alone***. Further, it is insufficient to simply show that a procedural risk is low, or zero, even if that was universally possible (see below).

### Medical Intervention

**Figure 3:**
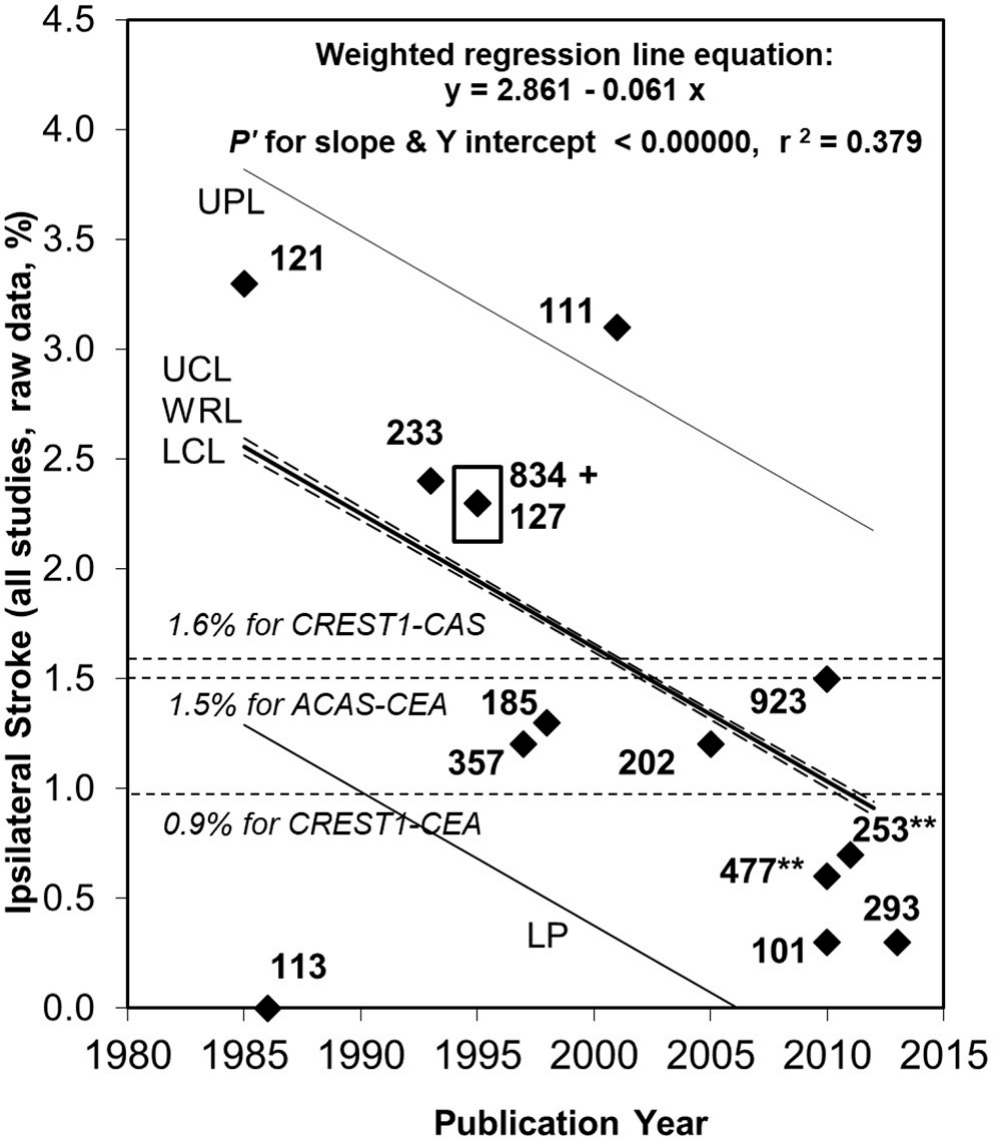
Ongoing fall in ipsilateral stroke rate in patients with ≥50% asymptomatic carotid stenosis given medical intervention alone between 1984 and 2013 (2, 21). Sixty seven percentage relative (or 1.7% absolute) fall in the reported average annual ipsilateral stroke rate in patients with ≥50% ACS given medical intervention alone from 1984 to 2013. Fifty six percentage relative fall since ACAS was published in 1995 with 834 medically treated patients, ACAS result highlighted by a black box (35). Black diamonds are study results with corresponding sample sizes. As in 2009, the Ryan-Holm stepdown Bonferroni correction was made for multiple comparisons (converting raw *P*-values to *P'*-values, SYSTAT 13, SYSTAT Software Inc) (21). UPL and LPL, respectively, = upper and lower 95% prediction limits for new population rate estimates; UCL and LCL (dashed lines), respectively = upper and lower 95% confidence limits for the population regression line; WRL, weighted regression line; ACAS, Asymptomatic Carotid Atherosclerosis Study (35); CREST-1, Carotid Revascularization Endarterectomy vs. Stenting Trial (65). **Indicates two studies including a small minority with remote ipsilateral stroke/TIA at baseline (94, 95). These were included in this analysis because the regression line was not appreciably different with (y = 2.861–0.061x, *P'* for slope and y intercept < 0.00000 and *r*^2^ = 0.379) (2) or without them (y = 2.720–0.051x, *P'* for slope and y intercept < 0.00000 and *r*^2^ = 0.275) (12).

It is now well-recognized that the stroke prevention benefit of medical intervention alone (risk factor identification, lifestyle coaching or healthy lifestyle habits and appropriate medication) has improved significantly over the last 3–4 decades in both ***asymptomatic and*** ***symptomatic patients with advanced carotid stenosis*** (13, 21–28). Indication of this improvement in relation to asymptomatic carotid stenosis patients was first published in 2007 and was then confirmed in more detail and by several independent groups across multiple countries (13, 21–25). Latest reported average annual ipsilateral stroke rates associated with advanced asymptomatic carotid stenosis patients treated with medical intervention alone approximate 0.8%. This is lower than with either CEA or CAS in previous randomized trials (see Figure 3, Table 4) (2, 12). Further, the most recently reported quality measurements of stroke rate with medical intervention alone were published around 2013 (2, 12). These latest studies likely underestimate what can be now achieved because medical intervention has continued to improve since they were performed and published.

Furthermore, recent studies have shown that aggressive medical intervention in specialized stroke/TIA clinics can dramatically reduce the early risk of recurrent cerebral ischaemic events in symptomatic patients awaiting CEA or CAS (26–28). These studies include a 2021 comparison of stroke rates among symptomatic patients awaiting a carotid procedure in three older randomized trials of CEA vs. medical intervention (recruitment between 1981 and 1996) and four more recent randomized trials of CEA vs. CAS (recruitment between 2000 and 2008). This analysis showed that symptomatic patients enrolled in the more recent trials had a lower rate of stroke after randomization than patients in the older trials (28). Improvements in medical intervention have also been demonstrated by ***better outcomes in other populations***, including symptomatic patients with intracranial arterial disease in the SAMMPRIS Trial (21, 96, 97).

Medical intervention consists of the diagnosis and management of leading arterial disease risk factors, including hypertension, blood lipids, diabetes, tobacco smoking, atrial fibrillation, physical inactivity, sleep disorders, and excessive weight and alcohol consumption. The nature of the medical intervention received in previous studies of carotid stenosis patients most likely reflected common practice at the time. However, this information was not reported or, at best, it was only partially reported (2, 12, 21). This lack of reporting probably reflects a general under-appreciation of the value of medical intervention at the time. In addition, knowledge regarding how to best reduce arterial disease risk through medical intervention has evolved over decades and across many specialties, causing confusion and uncertainty (see below).

We are preparing an in-depth, multi-expert review of current best medical intervention for prevention of arterial disease complications with a focus on carotid stenosis patients. This will be submitted for publication shortly. Highlights include the importance of separating carotid stenosis patients into those who are completely asymptomatic with respect to arterial disease (in the absence of another indication, they do not need antiplatelet medication) (98) from those who have been symptomatic. Best use of antiplatelet and anticoagulant drugs is also heavily dependent on how long ago a patient was symptomatic, what kind of symptoms they had, and whether or not they have atrial fibrillation and/or metallic heart valves (99–109).

Generally speaking, the threshold for diagnosing the leading arterial disease risk factors has lowered over the last 3–4 decades (21). Target blood pressure and cholesterol levels have lowered and now tend to be influenced by the patient's overall risk factor profile rather than using a “one target fits all” approach. For example, some now recommend a target low-density lipoprotein (LDL) cholesterol for asymptomatic or symptomatic persons with carotid stenosis of 1.4 mmol/L (55 mg/dL) or less, or at least 50% lower compared to baseline (110). However, the lower the LDL, the lower the risk of arterial disease complications (111–116). For every 1 mmol/L reduction in LDL, there is an expected overall reduction in the absolute average annual rate of major arterial disease complications of 0.8% (a relative risk reduction of ~20%) and 0.2% reduction in the absolute average annual rate of all-cause mortality (a relative risk reduction of ~10%) (111, 112). There are no known risks of lowering LDL per say. There are similar benefits for men and women (112). Serious adverse reactions from statins and ezetimibe are uncommon (110).

A general definition of hypertension >140/90 prevails in Australian and European guidelines (117–119). Meanwhile, >130/80 is now used as the definition of hypertension in the USA (120). Benefits are proportional to the degree of blood pressure lowering, rather than class of drug used (121). Overall, meta-analyses have shown that for every 10 mm Hg reduction in systolic blood pressure, there is an expected 1.9% absolute (20% relative) risk reduction in major cardiovascular events (fatal and non-fatal myocardial infarction, sudden cardiac death, revascularisation, fatal and non-fatal stroke, fatal and non-fatal heart failure) over the next few years or so (122, 123).

## Current Best Management Of Carotid Stenosis Patients

### Patients With Asymptomatic Carotid Stenosis

It can now be estimated that ~0% of low/average surgical risk patients with advanced asymptomatic carotid stenosis (like those recruited into ACAS or ACST-1 and included in meta-analyses) (2, 12, 35, 37) could now overall benefit from a carotid artery procedure if they are receiving current best practice medical intervention. This conclusion is derived from the following observations:

*i*. ***The average annual ipsilateral stroke rate was*** **~*****0.8%*** with medical intervention alone in the most recently published quality studies (2, 12, 21).
*ii*. ***About half the strokes occurring in the distribution of 60–99% asymptomatic*** ***carotid stenosis are not actually due to the stenosis*** (3). Therefore, the average annual ipsilateral stroke rate due to carotid stenosis (based on the most recently published quality studies of patients given medical intervention alone) is about ***0.4%***.
*iii*. ***The average age of diagnosing 50–99% asymptomatic carotid stenosis was*** ***70 years*** in past quality studies of patients given medical intervention alone and their ***average survival was 10 years*** (2, 12, 21). Therefore, at most, about 4% (10 times 0.4%) will have an ipsilateral stroke due to carotid stenosis during their remaining life time following diagnosis and could possibly benefit from a carotid procedure were they to receive medical intervention to the standard in the most recently published studies (2, 12, 21).
*iv*. ***Medical intervention has improved*** since 2013. Therefore, the 4% estimate of the maximum proportion of average/low CEA risk patients with advanced asymptomatic carotid stenosis who could possibly benefit from a carotid procedure during their lifetime is probably an overestimate.
*v*. ***The procedural rate of stroke and death*** and other significant complications cannot always and everywhere be zero. This is expected to negate any possible overall routine practice benefit from carotid procedures in patients with asymptomatic carotid stenosis in the modern era, particularly given that routine practice carotid procedural outcomes are often, if not usually, worse outside trials (13, 42, 43).

The available evidence indicates that ***we have passed an era in which carotid artery*** ***procedures are likely to provide an overall benefit for this population***. Meanwhile, patients with a sufficiently high, average annual ipsilateral stroke risk (despite current best practice medical intervention) have not been identified. Such patients, if they exist, are rare. It is currently impractical and unethical to routinely screen to detect them if the purpose is to select individuals for carotid artery procedures (see below) (12). Therefore, the current best management of patients with advanced asymptomatic carotid stenosis is ***current best medical intervention alone***. It will remain that way unless at least one subgroup of this population is identified that benefits from the addition of a carotid artery procedure in appropriate randomized trials (which is unlikely), and the results of such randomized trials are at least as good in routine clinical practice.

### Symptomatic Patients With Carotid Stenosis

Limited information is available with respect to outcomes of symptomatic patients with advanced carotid stenosis managed with current standards of medical intervention alone. This aspect of stroke prevention has not been properly retested, evidently based on the continuing false assumption that medical intervention has not changed and that CEA (or even CAS or TCAR) are indicated and beneficial for patients. However, as mentioned above, it is known that recurrent stroke and TIA rates in symptomatic patients awaiting a carotid procedure have fallen significantly over time as medical intervention has improved (26–28). Improvements in medical intervention over the last 3–4 decades mean that all past randomized CEA trials are outdated. In addition, the widely used 6% 30-day peri-procedural stroke or death rate “standard” used in routine practice, and derived from these outdated randomized trials, is presently excessive (1). As mentioned above, it is likely that fewer symptomatic patient subgroups will now benefit from a carotid artery procedure and the time window for procedural benefit is probably shorter compared to when NASCET, ECST-1, and the VACS were conducted 3–4 decades ago (49).

Symptomatic patients with carotid stenosis, however, have a higher stroke risk and are more likely to benefit from a carotid artery procedure than asymptomatic carotid stenosis patients. Symptomatic patients receiving current best practice medical intervention alone are the priority for stroke risk stratification studies (see below). Only those with a sufficiently high residual average annual ipsilateral stroke rate should be considered for future randomized trials involving carotid procedures vs. current best medical intervention alone. As explained below, the average annual ipsilateral stroke rate should probably be in the order of at least 3–4% despite current best medical intervention before randomized procedural trials are considered (71).

In the meantime, all ***symptomatic patients with carotid stenosis should receive*** ***current best practice medical intervention as soon as possible. Expertly performed*** ***CEA*** (not CAS or TCAR) ***could be considered for those who fit the profile of*** ***one of the 3 subgroups which had an overall benefit from CEA in NASCET***, ***ECST and the VA309 (see above)***, so long as the 30-day stroke or death rate is “acceptable.” The definition of acceptable is unclear. However, it should certainly be <6% (49). Independently of the surgical team, the 30-day peri-operative stroke or death rate should be systematically measured and adjusted for patient risk factors wherever CEA is performed. This information should be available at the point-of-care to allow clinicians and patients to make properly informed decisions. In addition, patients should be advised that the information for supporting this combined medical-surgical approach is based on highly selected patient subgroups and outdated, randomized CEA trials conducted 30–40 years ago.

Therefore, ***if consent for a carotid procedure is truly “informed” in a symptomatic*** ***patient***, *several issues must be discussed with that patient and their carers:*

*i*. The option of current best practice medical intervention alone, given the lack of current randomized trial data with respect to CEA benefit, known improved outcomes with medical intervention alone since the VA309, NASCET and ECST, the modest overall benefit from CEA in those randomized trials (3.2%/year) and that most patients (for example, about ~74% with 70–99% stenosis) did not have an ipsilateral stroke during follow-up in those randomized trials (4).
*ii*. The subgroups of carotid stenosis patients included and excluded in the VA309, NASCET, and ECST and the subgroups shown to benefit and not benefit in those trials.
*iii*. Identifiers of a particularly poor chance of procedural benefit and a particularly increased risk of procedural harm, such as advanced age and major comorbidity.
*iv*. Overwhelming evidence that CAS is more dangerous than CEA, while any TCAR benefit is unproven.

## Common “Furphies” Regarding Carotid Artery Procedures

According to Wikipedia, a furphie is Australian slang for rumor, or an erroneous or improbable story, but usually claimed to be absolute fact. A furphie is generally heard first or second hand from a reputable source and, until discounted, is widely believed (see https://en.wikipedia.org/wiki/Furphy). This section provides an outline of five common furphies used to make CEA and CAS appear “similar” with respect to their outcomes (when they are not) or to make carotid procedures appear “indicated” (when medical intervention is the only currently proven effective treatment).

### Omitting the Peri-Procedural Risk Furphie

Proponents of this first furphie imply that CAS and CEA are similar in outcome after a “successful” carotid artery procedure and/or emphasize only the outcomes beyond the peri-procedural period (36, 37, 68, 80, 81). By “successful,” proponents generally infer a procedure not complicated by peri-procedural stroke or death. However, one cannot have a carotid artery procedure without the peri-procedural period and those who will have a peri-procedural stroke or death cannot be accurately predicted. It is inappropriate to compare procedures without including the peri-procedural period when making treatment decisions. As explained above, in every adequately powered randomized trial comparison, CAS was found to cause significantly more peri-procedural strokes (with or without deaths or clinically-defined myocardial infarctions) than CEA (12, 49, 56, 57). Stroke rate differences between CAS and CEA appear similar beyond that time. However, if the peri-procedural period is indeed included, the higher rate of stroke in patients who had CAS compared to CEA has been seen for as long as patients have been followed-up in randomized trials. This indicates that surviving patients who have peri-procedural stroke tend to live long term with their stroke (while those that die remain dead). Therefore, the harm caused by CAS in terms of stroke and death is durable. Short through to long term outcomes from CAS and CEA are *not* similar (124).

### The Heart Attack Furphie

Proponents of this furphie imply that CAS and CEA are “similar” because the risk of stroke with CAS is compensated by the risk of myocardial infarction with CEA (70, 80, 125). Across the randomized trials of CAS compared to CEA, where the 30-day rate of stroke, clinically-defined myocardial infarction and death were reported, overall CEA was associated with nearly twice as many clinically-defined myocardial infarctions. However, CAS was associated with twice as many peri-procedural strokes and 1.5 times as many peri-procedural deaths than CEA (12). Moreover, in these randomized trials during the 30-day peri-procedural period, strokes and deaths (most associated with CAS) were 5.4 times more common than clinically defined myocardial infarctions (12). This means that, overall, CAS was associated with 1.6 times more peri-procedural strokes, deaths and clinically-defined myocardial infarctions than CEA (12). These comparisons reached statistical significance with respect to stroke, death, and myocardial infarction in symptomatic patients in meta-analyses of randomized trials (56, 57). However, there are still insufficient numbers of randomized asymptomatic patients studied to make an adequately powered comparison between CEA and CAS for this combined outcome measure. In summary, in randomized trials of CAS vs. CEA, CAS was overall associated with more peri-procedural stroke, death and myocardial infarction than CEA and the significantly elevated risk of stroke (or death) with CAS was not compensated by the smaller excess risk of myocardial infarction with CEA.

### The Most Severe Stroke Furphie

Proponents of this furphie claim or indicate that CAS and CEA are “similar” because disabling strokes [with modified Rankin Scale (mRS) score ≥3] in the ICSS, or other randomized trials, were about as common with each procedure (68, 77). Such reasoning is inappropriate. ***Firstly***, unless shown otherwise, all strokes should be assumed disabling. A modified Rankin score of 1 means able to carry out all usual activities of daily living despite some symptoms. A score of 2 means able to look after one's own activities of daily living without assistance, but unable to carry out all previous activities (126). For some patients, even this level of disability is likely be a significant infringement on their previous quality of life. In addition, the mRS only considers fundamental activities, mostly regarding mobility and self-care. It does not necessarily take into account more complex activities, such as the ability to return to one's previous employment.

***Secondly***, past randomized trials have been underpowered to exclude clinically significant differences in the rate of the most severe strokes associated with CAS and CEA. For example, as recently reported from ACST-2 (the largest randomized trial of CEA vs. CAS in ***asymptomatic/recently asymptomatic carotid stenosis patients***, with 3,625 total subjects), there were only 25 total 30-day peri-procedural strokes with a mRS score of 3–6 (13 with CAS and 12 with CEA, OR 1.09, 95% CI 0.46–2.61, *P* = 0.84) (68). The CI indicates that if the ACST-2 methodology was rolled out into routine practice, and thus involved a much larger sample size, it is within the realm of probability that CAS would cause up to 2.6 times as many of the most severe strokes as CEA. That excess harm associated with CAS would be clinically significant.

Meanwhile, the ICSS has been the largest randomized trial of CAS vs. CEA in ***symptomatic patients*** (1,713 total patients) (77). The ICSS was powered sufficiently to show that new diffusion-weighted lesions on magnetic resonance brain imaging (MRI) were more common after CAS (in an ICSS sub-study affecting 50% of the CAS patients and 17% of CEA patients, OR 5.2, 95% CI 2.8–9.8) (127). The ICSS was also sufficiently powered to show that *any* stroke (modified Rankin score of ≥1) by 5 years of follow-up was more common with CAS (affecting 15% of CAS patients and 10% of CEA patients, HR 1.7, 95% CI 1.3–2.3, *P* < 0.01) (77). However, the most severe strokes (with a modified Rankin score ≥3) occurred in only 6.5% of both CAS and CEA patients: HR 1.06, 95% CI 0.7–1.6 (77). The CI indicates that if the ICSS methodology was rolled out into routine practice, and thus involved a much larger sample size, it is within the realm of probability that CAS would cause up to 1.6 times as many of the most severe strokes as CEA. That excess harm associated with CAS would be clinically significant.

A trend with respect to more severe strokes with CAS compared to CEA was seen in meta-analyses of randomized trials of CAS vs. CEA in symptomatic patients (56, 57). Randomized trials have been underpowered with respect to comparing rates of severe stroke with CAS and CEA. Therefore, the procedural risk of severe stroke should not be used to justify CAS.

### The Late Disability Furphie

Proponents of this furphie indicate that CAS and CEA are “similar” because the prevalence of late disability (poor functional outcome) with both procedures is similar (77, 128). This comparison was derived from the ICSS and involved disability from any cause, not just disability due to strokes caused by carotid procedures. Disability from any cause reportedly affected ~60% of both CAS and CEA treated patients in the ICSS at 12 months post procedure and ~70% of both groups by 5 years post procedure (77, 128). Disability from any cause is common in this elderly population. Combining disability from stroke with all other causes of disability statistically camouflages, but does not remove, the excess disability caused by stroke caused by CAS compared to CEA. As above, the excess rate of stroke (and therefore stroke caused disability) associated with CAS compared to CEA is measurable for as long as patients have been followed-up in randomized trials. It is, inappropriate to discount this excess stroke associated disability from CAS by mixing it with disability from any cause.

### Limiting Carotid Procedures to “High Stroke Risk” Patients Furphie

This last featured furphie appears in recently published clinical guidelines for the management of patients with asymptomatic carotid stenosis (129–131). Proponents recommend CEA, or even CAS, for 50-99% asymptomatic stenosis patients if at least one of the following (or possibly other undefined) features, are present:

*i*. ***Silent infarct*** *on computed tomography brain imaging*
*ii*. ***Asymptomatic stenosis progression***
*iii*. ***Large plaque*** *area*
*iv*. ***Juxta-luminal black areas*** *on ultrasound*
*v*. ***Intraplaque hemorrhage*** *on MRI*
*vi*. ***Impaired cerebrovascular reserve***
*vii*. ***Plaque echolucency*** *on ultrasound*
*viii*. ***Transcranial embolic signals*** *with or without plaque echolucency*
*ix*. ***History of contralateral stroke or TIA***

However, for several reasons it is inappropriate to use these markers to justify carotid artery procedures in the routine management of asymptomatic carotid stenosis patients. ***Firstly***, not all of these markers have been shown to identify patients at higher than average ipsilateral stroke risk despite medical intervention (12). This includes detection of embolic signals (ES) with transcranial Doppler (TCD). Of the three studies investigating the association of ES detection and risk of subsequent stroke in asymptomatic carotid stenosis patients, two were flawed because nearly half the strokes that occurred during follow-up were in patients who already had an ipsilateral TIA during follow-up (94, 132, 133). Therefore, these were studies of mixed asymptomatic and symptomatic patients with carotid stenosis. The third study was negative (134).

***Secondly***, where measured, the average annual rates of ipsilateral stroke (12) and ipsilateral stroke or TIA (135) associated with such markers were generally too low to identify those likely to benefit from a carotid procedure. ***Thirdly***, no proposed stroke risk marker in carotid stenosis patients has been tested using current best practice medical intervention alone. Therefore, all past studies of these markers overestimate the current potential benefit of a carotid artery procedure (12). ***Fourthly***, most, if not all, proposed high plaque risk features are individually too common to identify the small proportion of patients who are now likely to benefit from a carotid artery procedure (a proportion which, as explained above, is close to zero, if such patients exist) (12, 135).

***Moreover***, as a group, proposed markers of high stroke risk may be used to cover just about any asymptomatic carotid stenosis patient, particularly when not all markers that may confer increased stroke risk are stipulated in guidelines (12, 130). Problems related to the lack of specificity of proposed stroke risk markers are compounded by guideline writers not providing reproducible definitions (129–131). For example, ~10% of asymptomatic carotid stenosis patients will have at least 1–2 ipsilateral middle cerebral artery ES detected after 1–2 h of TCD monitoring (133). However, this proportion increases to over 60% with more frequent monitoring (133). By way of another example, at least some degree of ultrasound echolucency is very common in 60–99% asymptomatic carotid stenosis plaques, reported in at least 63% of carotid duplex studies in the “ASED Study” (136). Finally, no proposed marker of high stroke risk in asymptomatic carotid stenosis patients has been tested in randomized trials of CEA vs. current best practice medical intervention alone (12).

Another common mis-justification for procedural intervention for asymptomatic carotid stenosis is ***the presence of 80–99% (rather than 50–79%) stenosis*** (137, 138). However, past research has shown that the average annual ipsilateral stroke rate associated with 50–80% and 80–99% (using NASCET or unspecified criteria) is small (in the order of 1 and 3%, respectively) (139, 140). Such rates are particularly low considering we can expect to significantly lower these rates with current best, intensive medical intervention alone, perhaps to 0.5–1.5% or less (12). Meanwhile, the ACAS is still the dominant trial with respect to showing a potential routine practice procedural benefit in patients with asymptomatic carotid stenosis (35). Following on from ACAS results for patients given medical intervention alone, a population of patients with asymptomatic carotid stenosis with an average annual ipsilateral stroke rate of at least 2–3%, despite current best medical intervention alone, should be sought before a carotid artery procedure might be considered to provide additional benefit (35, 71). However, in order to provide a buffer against net patient harm, an average annual ipsilateral stroke rate over 3–4% is probably more appropriate given that 30-day procedural stroke and death rates in routine practice are often, if not usually, higher than in randomized trials (12, 42, 43, 71).

In summary, using proposed “high stroke risk” markers to select asymptomatic patients for carotid procedures in routine practice will continue the widespread use of risky, costly and unnecessary carotid procedures that started decades ago (1, 12, 29). As mentioned, symptomatic patients with carotid stenosis are much more likely to benefit from a carotid procedure combined with current best medical intervention. Therefore, if the goal is to select patients who will benefit from a carotid artery procedure, ***symptomatic patients should be the*** ***priority for stroke risk stratification studies using the above markers and perhaps others***. Only those with sufficiently high residual ipsilateral stroke rate, despite current best medical intervention, should be considered for randomized trials of current best practice medical intervention with or without an additional best practice carotid artery procedure.

## Ongoing Randomized Trials Involving Carotid Procedures

Currently three randomized trials involving carotid artery procedures (CEA and CAS) are known to be underway or are planned. They each have medical-intervention-only-arm (“ECST-2,” “CREST-2,” and “ACTRIS”). None of these trials is likely to identify a procedural indication for carotid stenosis patients in current routine practice.

***ECST-2*** is the only randomized trial that involves symptomatic patients with carotid stenosis (http://s489637516.websitehome.co.uk/ECST2/index2.htm). However, only those who are symptomatic and considered at low stroke risk on medical intervention are being randomized to medical intervention with or without CEA or CAS. Hence, outcomes are likely to be similar in the treatment arms, or possibly worse with a carotid procedure. In ***ECST-2 and CREST-2*** (https://clinicaltrials.gov/ct2/show/NCT02089217) patients with asymptomatic carotid stenosis considered at low or average surgical risk (similar to those recruited into ACAS and the ACST-1) are being randomized to medical intervention alone with or without additional CEA or CAS. However, as explained above, such patients are unlikely to have an overall benefit from these procedures.

By contrast, in ***ACTRIS*** (https://clinicaltrials.gov/ct2/show/NCT02841098) patients with advanced asymptomatic (or recently asymptomatic) carotid stenosis considered at higher than average ipsilateral stroke risk despite medical intervention are being randomized to CEA or medical intervention alone. Markers of “high stroke risk” being used include history of contralateral TIA or ischemic stroke due to atherosclerotic carotid disease, silent brain infarction on MRI, predominantly echolucent plaque on ultrasound, the presence of transcranial Doppler (TCD)-detected embolic signals, intraplaque hemorrhage on MRI, TCD-measured impaired cerebral vasomotor reserve or rapid stenosis progression. However, it is likely ACTRIS will be underpowered because stroke rates in such patients have not first been measured with current best practice medical intervention alone. As mentioned, all past stroke risk stratification studies in asymptomatic carotid stenosis patients given medical intervention alone are outdated and overestimate any current potential procedural benefit. As explained above, only asymptomatic carotid stenosis patients with a sufficiently high residual average annual ipsilateral stroke rate (in the order of least 3–4%), despite current best medical intervention, should be recruited into randomized trials involving carotid artery procedures.

Meanwhile, the ***ACST-2*** investigators relatively recently randomized only asymptomatic (or recently asymptomatic) carotid stenosis patients to either CEA or CAS (https://clinicaltrials.gov/ct2/show/NCT00883402) (68). ACST-2 investigators may find support for the notion that CEA and/or CAS are relatively safe. However, as mentioned above, there was a trend for more stroke and peri-procedural death with CAS (68). Moreover, the ACST-2 investigators cannot establish a procedural indication over current best practice medical intervention alone because the trial did not include a medical-intervention-only treatment arm.

## What The Guidelines Say

In 2015 we demonstrated many ways in which “evidence-based” guidelines for carotid disease management encourage overuse of so-called carotid artery “revascularization” procedures (1). We found 34 guidelines published between 2008 and 2015, from 23 different regions or countries and written in six languages with recommendations regarding the use of CEA and/or CAS in relation to symptomatic and/or asymptomatic patients. Many of these guidelines have not been reiterated. With respect to **“*average surgical risk” patients*** ***with 50–99% asymptomatic carotid stenosis***, 96% (24/25) of applicable guidelines endorsed CEA and 63% (17/27) endorsed CAS by recommending, respectively, that these procedures should or could be done. The endorsements were given despite no evidence of procedural benefit over contemporary medical intervention, underpowered randomized trial comparisons of CAS vs. CEA (with safety trends against CAS) and known clinically significant procedural risks (1). In addition, 48% (13/27) of the applicable guidelines endorsed CAS for patients with asymptomatic carotid stenosis ***considered at “high surgical risk”*** due to arterial anatomy, comorbidities or undefined reasons. This is despite safety trends against CAS, not measuring outcomes with any standard of medical intervention alone and the likelihood that many such patients would not live long enough to benefit from a carotid artery procedure (1, 141–144).

Our critical comparative audit of worldwide guidelines for carotid stenosis management also revealed that 100% (31/31) of applicable guidelines endorsed CEA for **“*average*** ***surgical risk” symptomatic patients with 50–69% or 70–99% carotid stenosis***. Just over half the guidelines [18/33 (55%) and 19/33 (58%), respectively], endorsed CAS for both moderate and severe carotid stenosis in average surgical risk symptomatic patients. In addition, 82% (27/33) of the applicable guidelines endorsed CAS for symptomatic patients ***considered at “high-CEA-risk”*** because of their vascular anatomy, comorbidities of undefined reasons. These endorsements were given despite no evidence of procedural benefit over contemporary medical intervention, clinically significant procedural risks (especially for CAS) and direct evidence that many “high-surgical-risk” patients will not live long enough to benefit from a carotid artery procedure (1, 141–144).

Other ways in which guidelines over-encourage carotid artery “revascularization” procedures include not limiting procedural recommendations to subgroups shown to overall benefit from CEA compared to medical intervention alone in past randomized trials, not acknowledging that all past randomized trials involving CEA are outdated, and not clearly defining target populations and standards with respect to the 30-day peri-procedural rate of stroke and death (1). In addition, guidelines often omit recommendations for medical (non-invasive) interventions which are currently proven to reduce the risk of stroke and other arterial disease complications (1).

We are performing an updated critical comparative audit of guidelines regarding carotid “revascularization” procedures. Unfortunately, the ***procedural biases mentioned above*** ***are still common***, including in the most recently published guidelines (145–147). At least with respect to asymptomatic carotid stenosis patients, guidelines from Australia and Denmark discourage CEA and CAS or screening (148–150). Meanwhile, guidelines from the UK and USA were published which came at least part way in overcoming procedural bias in recommendations for asymptomatic carotid stenosis patients (151–153). However, unfortunately the improved UK guidelines were replaced by guidelines which omitted recommendations for asymptomatic carotid stenosis patients (154). Improved guidelines for symptomatic patients are even slower to emerge.

## How Times Are Changing For The Better

### Existing Changes

***The good news is that stroke prevention has become much cheaper, more*** ***effective and less invasive over the last 3–4 decades*** (13, 21–28, 96, 97). This is testimony to the success of researchers, public health campaigners, policy advisors, educators, and the general public. Now it is clear that the individual has the most power to prevent their own stroke. Improvements in medical intervention provide hope not just for patients with carotid artery disease but for everyone. This is because medical intervention reduces the risk of all arterial disease complications in all at risk.

As indicated above, improved medical intervention in carotid stenosis patients has prompted some guideline updates and new randomized trials of carotid procedures to include a medical-intervention-alone arm (when previously only CEA and CAS were compared). There have been policy decisions that protect the public from unnecessary, risky and expensive carotid artery procedures (61, 62). Our successful campaign to advise US Medicare not to expand CAS reimbursement indications in 2012 led to the establishment of the Faculty Advocating Collaborative and Thoughtful Carotid Artery Treatments (FACTCATs) (61, 62). This is a growing group with currently over 365 clinicians and scientists of all career stages with an interest in stroke prevention. FACTCATs communicate and collaborate via simultaneous group email. Members with diverse views are welcomed and there is ongoing encouragement that opinions are substantiated by factual scientific evidence. This group, and the FACTCATs website (see https://factcats.org/), are effective and novel ways to provide large-scale education to professionals and the public. Moreover, rates of carotid procedures are falling in the USA and UK (155–158). Additionally, to drive further improvement in the field, two important new initiatives (or “emerging changes”) are underway, as will be explained next.

### Emerging Changes

#### The First “Evidence-True” Guideline for Carotid Artery Disease Management

The flaws in existing carotid disease guidelines are being used to define methods to ***maximize guideline objectivity and focus on optimizing patient outcomes***. These criteria will be utilized in the first “evidence-true” guideline for carotid artery disease management (159). These novel methods could be applied generally to clinical practice guidelines no matter the health condition. This new carotid disease guideline is being produced under the auspices of the International Union of Angiology (IUA, Abbott et al., in preparation, see: https://factcats.org/). The IUA provides focus for scientific endeavor across all specialties involved in vascular disease (see https://www.angiology.org). Existing good practice guideline methodology will be retained, including multi-specialty and consumer contribution (160, 161). ***Novel, generalizable methods include:***

*i*. ***Limiting guideline endorsements for interventions to subgroups that*** ***benefited in relevant trials*** and clearly distinguishing subgroups included and not included in such trials.
*ii*. ***Not treating subgroups the same*** (such as men and women) if the evidence clearly shows significantly different outcomes with the same treatment.
*iii*. ***Acknowledging when trials are outdated***, such as all past trials of CEA.
*iv*. ***Acknowledging when interventions cause excess harm***, such as CAS.
*v*. ***Including recommendations for proven medical interventions***, not just for procedures.
*vi*. ***Not automatically ranking randomized trial data as best, even if outdated or*** ***biased***. Remarkably, evidence (research) appraisal is often lacking in guidelines, as well as guidelines for creating guidelines (1, 160, 162). However, the ***GRADE system*** is sometimes used, where evidence based on randomized trials begins as the highest quality evidence (163–165). Movement down levels is possible. However, the mechanism of movement is unclear, subjective and not inherently mandated (163–165). We have not noticed demotion of randomized trial evidence in any guidelines we have reviewed (1).

To facilitate objectivity, I have created ***a novel, 12-point scoring system with respect to*** ***point-of-care evidence applicability***. This new “***pertinence score***” is a generic checklist of fundamental criteria required for research to be relevant to particular patients at their point-of-care. Factors to be considered include whether or not randomization is required for treatment decisions. For example, a randomized trial is not required to accurately measure a very low stroke rate with medical intervention alone and show, therefore, that a carotid procedure is not indicated. Other scored factors include whether or not study methods are current and key definitions are reproducible.

#### The CASCOM Study

Improvements in the effectiveness of medical intervention alone have made a welcome and dramatic impact on the management of patients with arterial disease. The highest priority in arterial disease is to properly document the nature of current best practice medical intervention and measure its impact in all kinds of patients, including those with carotid stenosis of any degree of severity. To help address this need, my collaborators and I are taking steps to establish the ***Carotid Stenosis Management During the COVID-19 Era with Best*** ***Medical Intervention Alone (CASCOM) Study***. The CASCOM Study is a prospective study of multiple cohorts of asymptomatic and symptomatic patients with carotid stenosis who do not undergo a “revascualrisation” procedure for any reason, including lack of resources caused by the coronavirus pandemic, situations of unproven procedural benefit, anticipated procedural futility or net harm or patient refusal. Hence, we will study patients for whom carotid procedures are not possible or considered unethical (see http://factcats.org/opportunities.php and https://www.anzctr.org.au/ACTRN12621001604897p.aspx).

To save clinician-researcher time, and therefore make the CASCOM Study feasible, we first need to enable the **“*Brainy Medicine” approach to healthcare***. This is where quality research data is produced as a by-product of usual patient reporting in clinical practice. Enabling Brainy Medicine involves innovative software programming and teamwork. We will separate CASCOM Study participants into those who would, and would not, have been eligible for past randomized trials of CEA versus medical intervention alone. Patients considered “eligible” for those randomized trials will be used for hypothesis testing. Given the available evidence (summarized above) we expect at least a 50% lowering of stroke rates with medical intervention alone in the CASCOM Study compared to that seen in past randomized trials of medical intervention with or without CEA. In addition, we plan to study patients in the latter randomized trial “ineligible” category and report their ipsilateral stroke rate over 5 years of follow-up. In contrast to the randomized trials of CEA or CAS presently being conducted, in CASCOM we will study low-, average- and high-surgical-risk patients. We will also independently check the quality of the medical intervention being used in other trials.

As mentioned above, we are preparing an in-depth, multi-expert review of current best medical intervention for prevention of arterial disease complications with a focus on carotid stenosis patients. This will be submitted for publication shortly and used to define standards for medical intervention in the CASCOM Study. This review also aims to address confusion over what constitutes current best medical intervention (49). The following are a few examples of confused issues to be addressed:

- ***Atrial fibrillation (AF)*** guidelines do not always limit anticoagulation recommendations to those with ***recent*** AND recurrent or persistent AF (166). Risk according to AF type is heterogeneous (167). Using anticoagulation beyond patient types who benefited in trials is over-treatment and exposes patients to a life-threatening bleeding risk (about 2–3%/year) without proven benefit.
- Major inconsistencies exist in guideline risk calculator recommendations on when to start ***lipid lowering therapy*** in primary prevention (168), implying error in evidence interpretation.
- “FACTCATS” discussions reveal variability in expert use of treatments, such as ***antiplatelet therapy*** for primary prevention (in asymptomatic patients). Some still inappropriately recommend it based on outdated trials (98).
- The Australian Pharmaceutical Benefits Scheme (PBS) has just announced subsidized use of ***low-dose rivaroxaban and aspirin*** in selected patients with asymptomatic carotid stenosis (see, https://www.pbs.gov.au/medicine/item/12192Q-12197Y|) However, there is no randomized trial evidence of significant benefit in patients with carotid stenosis (169). This PBS decision is likely to encourage exposure of patients to an unjustified risk of ***anticoagulant***-linked bleeding.

## Current Priorities And The Way Ahead

Among the highest research priorities now is to properly ***document the nature of current*** ***best practice medical intervention*** for prevention of stroke and other arterial disease complications and ***measure its impact***. Studies should include risk stratification with the goal of identifying asymptomatic and symptomatic patients likely to benefit from more aggressive or specialized medical and/or procedural interventions (41). Trials of new (and especially relatively high risk and/or expensive) interventions depend upon us first determining what can be achieved with the available effective interventions. Markers having the most promise with respect to carotid stenosis ***stroke risk*** ***stratification*** include standardized ultrasound characterization of plaque morphology combined with degree of stenosis and clinical features, MRI evidence of intraplaque hemorrhage and silent progressive stenosis toward occlusion (12, 139, 170, 171). As indicated above, it is more likely that such risk markers will identify symptomatic patients likely to benefit from a carotid artery procedure than asymptomatic carotid stenosis patients. Only patients with sufficiently high ipsilateral stroke rate, despite current best practice medical intervention alone, should be considered for randomized trials involving a carotid artery procedure. Meanwhile, we need to ***improve access*** ***to current best practice medical intervention***, establish ways to ***systematically*** ***measure outcomes from routine practice*** services using electronic health records and remove access to (or payment of) harmful and useless interventions (156, 159, 172). Resources saved should be redirected to better support effective services and more research (49).

Like it or not, and in ***answer to the “big question,”*** there is no current randomized trial evidence that a carotid artery procedure provides an additional stroke risk reduction benefit compared to current best practice medical intervention alone in any subgroup of carotid stenosis patients. To establish a current routine practice indication for a carotid artery procedure, at least one patient subgroup must be shown to benefit overall. This demonstration of overall patient benefit will depend on:

- Properly conducted ***risk stratification studies using current best practice*** ***medical intervention alone*** (to identify those with sufficiently high residual ipsilateral stroke rate), then
- ***Randomized trials*** of current best practice medical intervention alone with and without the addition of a best practice carotid artery procedure (only in those with sufficiently high ipsilateral stroke rate despite current best practice medical intervention alone) then
- Ensuring that favored randomized trial methods and results are ***duplicated at the*** ***point of care***.
- ***Acknowledging and addressing biases*** which continue to drive inappropriate carotid procedures, so we can provide only what is known to be effective treatment and perform the required research (1, 173, 174).

***All things considered, we require a worldwide revolution in medical training, public*** ***education and resource allocation***.

There is no current evidence that ***screening for asymptomatic carotid stenosis*** is beneficial for patients. Screening cannot be recommended if the intention is to identify patients for carotid artery procedures given the inherent procedural risks and no current evidence of procedural benefit. It is known that carotid imaging improves patient motivation to adhere to medical intervention and improves risk stratification compared to using clinically defined risk factors alone (175, 176). However, studies are required to determine if, and how, arterial imaging improves patient outcomes compared to managing clinically-defined risk factors alone (176). Currently, ***screening for carotid stenosis in stroke or TIA patients*** can only be justified for the 3 groups shown to have benefited overall in NASCET, ECST and the VA309 (as outlined above). However, this is while acknowledging that all past randomized trials of CEA vs. medical intervention alone are outdated and there is an urgent need for risk stratification studies and randomized procedural trials in symptomatic patients with sufficient residual ipsilateral stroke risk despite current optimal medical intervention.

Some cite or imply “improved,” “acceptable,” “comparable,” “within guideline” standards or “similar” procedural outcomes, and “low risk” from CEA or CAS (such as in-hospital [rather than 30-day periprocedural] stroke or death rates below 2% for asymptomatic carotid stenosis patients) as sufficient justification for continuing carotid artery procedures (42, 65, 68, 145, 177–179). However, this is inadequate and inappropriate. ***A*** ***procedure must be shown to provide a clinically significant net patient benefit (that*** ***outweighs procedural risk)*** compared to current best practice medical intervention alone. The likelihood that a particular patient will overall benefit from, or be harmed by, a carotid artery at their point of care is the most important issue when advising patients with carotid stenosis. This is more important than other considerations, such as culture and ethnicity (180, 181). Further, patient preference is a prerequisite for any intervention (182). However, patient preference strongly depends on the way relevant information is presented (or omitted). This has already been demonstrated regarding the topic of asymptomatic carotid stenosis (183).

***Patients should be advised that medical intervention is very powerful and the most*** ***effective proven way of reducing their arterial disease risk***. Further, in at least the majority of patients, it is unlikely that a lack of adherence with current best practice medical intervention can be fully compensated by a carotid artery procedure, given the high level of effectiveness of medical intervention, the limited procedural benefit in previous randomized trials and the inherent procedural risks, particularly in routine practice.

Finally, it is essential everyone (including clinicians, patients, and carers) keep in mind that ***we cannot prevent all strokes or other arterial disease complications. The best that*** ***can be done is to choose the management strategy most likely to give the best*** ***chance of a favorable patient outcome*** (taking patient, intervention and service provider factors into account). Meanwhile, it is important to continue efforts to improve management strategies and improve patient access to the most effective management strategies. In the case of arterial disease prevention, medical intervention (lifestyle coaching/healthy lifestyle habits and appropriate use of medication) is the most effective strategy. ***Using the principle of “first do no harm,” medical*** ***intervention is the gold standard by which invasive interventions must always be*** ***compared*** (184).

## Author's Note

AA is the founding member of the Faculty Advocating Collaborative and Thoughtful Carotid Artery Treatments (FACTCATS, see https://factcats.org/). FACTCATs have a shared goal of optimizing stroke prevention. By design, clinicians and scientists of diverse views are encouraged to be FACTCATs in this online environment which encourages substantiation of opinion with scientific evidence. The views of particular FACTCATs do not necessarily reflect the views of other FACTCATs.

## Author Contributions

The author confirms being the sole contributor of this work and has approved it for publication.

## Funding

AA was part-funded by an NHMRC grant (APP ID: APP1168295) at the time of creating this manuscript.

## Conflict of Interest

The author declares that the research was conducted in the absence of any commercial or financial relationships that could be construed as a potential conflict of interest.

## Publisher's Note

All claims expressed in this article are solely those of the authors and do not necessarily represent those of their affiliated organizations, or those of the publisher, the editors and the reviewers. Any product that may be evaluated in this article, or claim that may be made by its manufacturer, is not guaranteed or endorsed by the publisher.
